# The Use and Comparative Merits of Bichloride of Methylene as an Anæsthetic

**Published:** 1874-08

**Authors:** Benj. F. Dawson

**Affiliations:** New York.


### SELECTED ARTICLES.

### ARTICLE III.

### *The Use and Comparative Merits of the Bichloride of Methylene as an Anæsthetic.*

BY BENJ. F. DAWSON, M. D., NEW YOK.

(Read before the New York Medical Library and Journal Association, April 17th, 1874.

While in London during June, 1872, I had the pleasure of being invited by T. Spencer Wells to assist him in a case of ovariotomy, in which he wished to test the merits of my clamp for the pedicle.

Aside from the great skill of this distinguished surgeon, I was much attracted by the appearance and behavior of the patient under the anæsthetic, as well as by the manner in which the latter was administered. In appearance the patient seemed to be in a natural sleep; neither her color nor respiration indicating that she was in a state of unnatural unconsciousness.

At the conclusion of the operation I was still more surprised to see how rapidly she recovered consciousness, not more than two minutes elapsing from the painless introduction of the last stitch and complete consciousness, shown by her opening her eyes and addressing Mr. Wells. There was no complaint of either nausea or headache.

On inquiry, Mr. Wells informed me that the anæsthetic used was the bichloride of methylene, or chloromethyl, as it is commonly called, and that he had used it for some time in preference to all other anæsthetics, for the reasons I will hereafter mention.

On my return home I brought with me a supply of chloromethyl, and the apparatus by which it is generally administered, and for the past two years I have used it, for myself and others, in both brief and prolonged operations.

The composition of bichloride of methylene is CH. CL. It is a clear liquid, with an odor somewhat sweeter than chloroform, boils at 88° Fahrenheit—54° lower than that required by chloroform—contains one atom more of hydrogen and one less of chlorine than the latter.

It resembles ether in being inflammable in air, but is more volatile, of more agreeable odor, and less irritating to the air-passages.

Chloromethyl was introduced as a general anæsthetic, in 1867, by Dr. B. W. Richardson, of London, who administered it for the first time in October of that year, in a case of ovariotomy performed by Mr. Wells, having previously subjected himself to its effects.

Since 1867 it has been extensively used in many of the largest hospitals of Europe, as well as in the practice of many English surgeons, by many of whom, notably Mr. Wells, it is considered superior to all other anæsthetic agents, with the exception that it is very expensive, from the fact that it is difficult to make it well, and still more difficult to preserve, owing to its liability to deterioration by exposure to light and air.

Like chloroform and sulphuric ether, it may be administered on a handkerchief or towel, or with any of the inhalers in general use; but from the peculiarity of being impaired by light and air, and its great volatility, it is best administered by such means as will guard against its being thus affected. Several apparatuses have been devised to accomplish this end, but none have been so generally endorsed as that known as Dr. Junker's, and which is the one preferred by Mr. Wells. Administered by this apparatus, a patient may be kept in a state of perfect unconsciousness throughout a prolonged operation; scarcely any of the vapor escapes into the room; neither the surgeon nor the assistants are affected by it; the light is excluded from the chloromethyl, and the quantity inspired by the patient is wholly within control of the one administering the anæsthetic.

The effects of bichloride of methylene are very similar to those of chloroform, but anæsthesia seems to be much more readily induced by it, and is decidedly more easily recovered from. In fact, its action seems to combine the best properties of chloroform and ether. According to Mr. P. Marshall, who reported the use of it in five cases before the Medical Society of London, in 1867, the time required to put the patient under its complete influence was from 3½ to 7 min., and the quantity used from 2 to 7 drachms.

In 126 cases in which it was given by Mr. Richard Rendal, the ages varying from 6 months to 70 years, anæsthesia was produced in 30 seconds in 18 cases; in 60 seconds in 70; in 2 minutes in 25; in 3 minutes in 5; in 5 min. in 3; in 9 min. in 2. Of these 123 cases 50 recovered in 1 min.; 23 recovered in 2 min.; 9 recovered in 3 min.; 28 recovered in 5 min.; 11 recovered in 10 minutes.

In my own experience, as shown by the records of cases, anæsthesia was invariably induced within 2 minutes, the longest being 8 minutes—one case which had previously resisted taking ether. Recovery was correspondingly quick.

Its effects being much more rapid, from its great volatility, than those of chloroform, anæsthesia is also induced much more quietly, there being, according to the testimony of those who have used it extensively, a marked absence of mental and muscular excitement or rigidity.

Its effects upon the pulse are, at the outset, to decidedly stimulate and strengthen it; but it soon returns to about its normal state, and never in my experience, as will be shown further on, has fallen much below its normal rapidity or strength. This statement is also supported by the testimony of Mr Richard Rendal, who attributes its safety to this stimulating action on the heart, and its rapid elimination, as shown by the rapidity with which patients recover from its effects.

Its effect upon the pulse in the five cases reported by Mr. Marshall, already referred to, were as follows: In three it ranged from 74 to 80; others offered a marked contrast, for in them it ranged from 114 to 120.

In 15 cases of my own, of which a record of the pulse was noted, the following was the lowest the pulse ranged: In three, 75; in five, 80; in three, 85; in two, 88; in one, 96; in one, 98.

Its effects upon the respiration are similar to chloroform. In no case have I seen other than full and quiet inspiration and expiration, and that these functions are properly performed is attested by the generally unaltered color of the skin and lips, which are also in some cases increased to a clear scarlet. This testimony is also supported by that of others especially Mr. Wells, who has used it more than 350 times, and who writes: "The patient very seldom becomes pale, sleeps quietly, and seldom has much bronchial irritation."

The nausea usually following recovery from ether and chloroform is of much less frequency and severity after chloromethyl. In regard to this point Spencer Wells also says: "It is quite true that it has the disadvantage of causing nausea and occasional sickness, but in my experience this is almost always the rule with chloroform, whereas with chloromethyl it is *certainly exceptional*." Mr. Rendal also states that "no sickness or headache follow unless the inhalation has been continued, or a second anæsthetic given to keep up the effects." In his 123 cases, already referred to, vomiting occurred in 15 only: 1 in 8—but in all of these latter it had been continued some time, and some had just been eating. In my own experience, in 31 cases in which I have administered it, nausea or vomiting ensued in but 6, in which latter it was administered for from 20 minutes to one hour, and for severe operations. I have never seen either nausea or headache ensue when it was administered for less than 10 minutes. And Dr. Thomas Bird, of Guy's Hospital, London, writing to me a few weeks ago, says: "As to nauseating effects, if the administration is PROLONGED, the effects are the same as with chloroform, except that the *sickness is not so prolonged.*"

As to the question whether chloromethyl is less dangerous than chloroform, or as safe as sulphuric ether, there is some uncertainty.

Mr. Rendal, in his article already referred to, says: " As regards safety, I may add that I have not had a fatal case." And Mr. Wells likewise writes that in over 350 cases in which chloromethyl had been administered for him by Drs. Richardson, Junker and Day, " in very few of these operations was the sensibility to pain maintained for less than 5 minutes—in a few it was kept up from 45 minutes to an hour or more—yet I have never been at all uneasy during the administration or from any subsequent effects fairly referable to it, whereas with chloroform I never felt quite at ease; and although I have never lost a patient during operation, I have three times had to resort to artificial respiration, and I have very often seen patients suffer so much from *chloroform vomiting*, that the result has been imperilled, and in some cases a fatal result has been in a great measure due to this vomiting. When I add that between April, 1870, and March, 1871, I had 31 successive cases of ovariotomy in private practice, without one death, and that the last 24 cases of the fifth hundred, including both hospital and private cases, all did well—every patient having recovered—it must be admitted ( as anæsthesia was complete in every case, not one patient having been conscious at any stage of the operation ) that the anæsthetic is a good one."

In the 31 cases in which I have administered it, 5 being cases of ovariotomy, and 10 prolonged and severe uterine operations, I have never seen a symptom that made me feel uneasy, and none that so often seem alarming in using chloroform. Indeed, so safe do I consider chloromethyl, when it is carefully given, that I have not hesitated to let others administer in my operations, whom I considered competent to judge of its effects, and Dr. P. B. Porter, who has given it for me eight times, as well as Drs. John C. Jay and F. H. Rankin, have had opportunity to watch its reliable action and evident greater safety over chloroform.

Death, however, has occurred during its administration, and, on looking through the literature of the subject, I have been able to find six cases recorded. In three of these the post-mortems show a large flabby heart; but though it may be proven to be more immediately dangerous than ether, yet the question can plausibly be asked, that if ether has not killed by its immediate anæsthetic effects, how many fatal results *after* operations have been due to its after effects?

Statistics kindly sent me by my friend Dr. Bird, of Guy's Hospital, give the following as the comparative death-rate of the anæsthetics now used:

| | |
|---|---|
| Ether, 1 death in - - - - | 23 000 |
| " and chloroform combined, 1 death in | 5,000 |
| Bichloride of methylene, 1 death in - - | 5,000 |
| Chloroform, - () - - | 3,000 |

Having briefly stated the effects and apparent advantages of the chloromethyl, I will briefly report a few of the most prolonged cases of the 31 of anæsthesia induced with it by myself, and thus show all the advantages claimed for it.

Case I.—Oct. 24th, 1871, I was asked to administer it by Dr. Emil Noeggerath, at the German Hospital, in a case of ovariotomy. Pulse rose at first to 100, but soon fell to 80, at which it continued strong during the *hour and a half* occupied by the operation. Respiration and color natural; but *one ounce* of methylene used. Anæsthesia was induced in 3 minutes, and recovered from in 12, the duration of the latter being due to her exhaustion from the great amount of blood lost.

Case II.—October 30th, 1871, was again requested by Dr. Noeggerath to give chloromethyl in a case of ovariotomy at the German Hospital. Anæsthesia complete in 2 minutes. Pulse, 105 at first, fell to 84, and continued at that rate, with but slight variations, quite strong. Respiration and color natural; recovery in 3 minutes; no nausea until evening, when vomiting occurred, as had been the case for 2 or 3 days before the operation, as is recorded in Dr. Noeggerath's report of the case to the Obstetrical Society. Operation lasted three quarters of an hour. ℨ v. methylene used.

CASE III.—Oct. 31st, 1871, gave methylene for Dr. Noeggerath, at German Hospital, in case of *ovariocentesis vaginalis.* Anæsthesia induced in 3 minutes. Pulse at outset rose to 95, but soon fell to 80, and continued strong at that rate during the operation, which lasted 20 minutes. Respiration and color normal. Recovered in less than 2½ minutes. No nausea and headache. ℨ iiiss. methylene used.

(These three cases will be found in the Transactions of the New York Obstetrical Society, reported in the *American Journal of Obstetrics* for Nov., 1872, and were witnessed by a number of our distinguished physicians.)

CASE IV.—Nov. 12th, 1872, was requested by Dr. T. A. Emmett to give methylene to a patient in the State Woman's Hospital, on whom he was to operate for vesico-vaginal fistula, and who had been exceedingly troublesome in previous attempts at anæsthesia. Was fully anæsthetized in 4½ minutes; made no resistance whatever. Pulse, 105 at first, soon fell to 80, at which it remained strong during the half hour occupied by the operation. Became conscious in less than 2 minutes; no nausea. When asked by Dr. Emmet how she felt, said "finely." But ℨ iij. of methylene used. Color and respiration natural.

CASE V.—Nov. 17th, 1872, was requested by Dr. Sims to give methylene to a lady on whom he was to operate for *carcinoma uteri.* Pulse rose at first to 112, but soon fell to 99 and 96, at which it remained during the hour and a quarter occupied by the operation. Was greatly excited at first, but soon breathed freely, and in 6 minutes was fully anæsthetized. Respiration and color natural. Lost considerable blood; recovered fully in 4 minutes. Some nausea, but nothing vomited. ʒ j. methylene used.

Case VI.—Nov. 20th, 1872, was again invited by Dr. Sims to give methylene to a case he has to operate on for *vaginismus.* Patient greatly excited and nervous, breathed badly at first. Pulse, 105 at outset, fell in a few minutes to 80, anæsthesia complete in 5½ minutes. Operation lasted 45 minutes; recovered consciousness in 3 minutes; some nausea and slight vomiting. ʒ vij. methylene used.

Case VII.—Nov. 23d, was invited by Dr. Sims to give methylene in a case of amputation of the *cervix uteri.* Pulse at outset 95, fell to 75, and continued at that rate; anæsthetized in 3 minutes. Color unusually bright and respiration free and full. Operation lasted 50 minutes; recovered consciousness in 2 minutes; very slight retching. ʒ iv. methylene used.

Case VIII.—Nov. 3d, 1873, gave chloromethyl to a patient on whom I was to operate for fissure of *cervex vteri.* Took it well, and in 4 minutes was perfectly anæsthetized. Pulse at first 100, fell to 85, at which it remained strong during the 45 minutes of operation. Respiration and color good. Came out of effects in 3 minutes; no nausea. ʒ vij. used.

Besides the above typical cases, which I have selected as those in which the chloromethyl was given for the longest time, I have also the records of others equally convincing as to the advantages of this anæsthetic. I have given it also for Dr. T. G. Thomas in ovariotomy, for Kammerer, Janvrin, and Hanks in other cases, and I am sure they will agree as to its apparent advantages.

The conclusions to be deduced from the foregoing remarks as to this anæsthetic are the following:

It is pleasanter than ether and chloroform in odor.

It is much more rapid in its effects than any other anæsthetic.

It induces no muscular excitement.

Recovery is more rapid and complete than from ether or chloroform.

Nausea and vomiting are rarely induced by it.

Its effect upon the circulation is not so depressing as chloroform, and respiration is much more regular and free.

Like chloroform, its effects are obtained with a small amount, hence it is much more portable.

In fact, I can offer no better praise of its advantages than that by Spencer Wells, who says: "Indeed, the patient has all the edvantages of complete anæsthesia, with fewer drawbacks than I have ever obtained by the use of any other anæsthetics."—*Medical Record.*

